# Microbiome–Immune Interaction in Pulmonary Arterial Hypertension: What Have We Missed?

**DOI:** 10.34133/research.0669

**Published:** 2025-04-09

**Authors:** Xin Zhou, Wen Tian, Shenbiao Gu, Marlene Rabinovitch, Mark R. Nicolls, Michael P. Snyder

**Affiliations:** ^1^ Department of Genetics, Stanford University School of Medicine, Stanford, CA, USA.; ^2^ Division of Pulmonary, Allergy, and Critical Care Medicine, Stanford University School of Medicine, Stanford, CA, USA.; ^3^ Veteran Affairs Palo Alto Health Care System, Palo Alto, CA, USA.; ^4^ Basic Science and Engineering (BASE) Initiative at the Betty Irene Moore Children’s Heart Center, Lucile Packard Children’s Hospital, Stanford University School of Medicine, Stanford, CA, USA.; ^5^ Stanford Cardiovascular Institute, Stanford University School of Medicine, Stanford, CA, USA.

## Abstract

Pulmonary arterial hypertension (PAH) is a devastating disease characterized by perivascular inflammation, immune dysregulation, and vascular remodeling. Recent studies have unveiled a potential link between the gut microbiome and PAH pathogenesis, suggesting that microbial dysbiosis and increased intestinal permeability may contribute to the inflammatory pathology in PAH and ultimately disease progression. This perspective highlights the emerging evidence of the role of leaky gut in PAH, the interplay between microbiota-induced immune responses, and the activation of endogenous retroviruses like human endogenous retrovirus K. Understanding these complex interactions opens new interdisciplinary avenues for research and therapeutic interventions, potentially transforming PAH management through microbiome-targeted strategies.

## Background

Despite recent clinical advances, pulmonary arterial hypertension (PAH) remains a progressive and life-threatening disease characterized by elevated blood pressure in pulmonary arteries, leading to right ventricular failure and increased morbidity and mortality. Clinically, PAH manifests with shortness of breath, fatigue, chest pain, and syncope, substantially impacting patients’ quality of life and survival. Although relatively rare, with an estimated prevalence of around 22.8 cases per million globally [1], PAH carries a poor prognosis despite recent advances in medical therapies.

PAH can arise in several clinical contexts: idiopathically, without an identifiable underlying cause (idiopathic PAH), or in association with exposure to specific drugs, certain toxins, or other inflammatory diseases (associated PAH). Familial PAH has been closely associated with germline heterozygous mutations in genes within the bone morphogenetic protein/transforming growth factor-beta pathways. Loss-of-function mutations in the gene encoding bone morphogenetic protein receptor type 2 (BMPR2) are identified in approximately 70% of familial PAH cases and 20% of idiopathic PAH cases [2], underscoring its important contribution to the disease (BMPR2) [3].

## Inflammation in PAH

Emerging evidence suggests that PAH is a complex multifactorial disease influenced by inflammation and immune dysregulation. Regardless of the underlying etiology, elevated levels of pro-inflammatory cytokines were documented in patients with all forms of PAH. Increased concentrations of interleukin (IL)-6, IL-1β, and tumor necrosis factor-alpha, as well as enhanced perivascular infiltrates, correlate with worse clinical outcome and prognosis [4,5]. Inflammatory mediators (e.g., leukotriene B4) also promote the apoptosis and transformation of pulmonary arterial endothelial cells and the proliferation and activation of pulmonary arterial smooth muscle cells, critical processes implicated in vascular remodeling [6–10].

Abnormal activities of the CD4^+^CD25^hi^Forkhead box P3 (FOXP3)^+^ regulatory T cells (Tregs) are implicated in PAH and its associated conditions and may explain the features of chronic vascular inflammation and immune dysregulation in disease [11]. Additionally, an increased number of IL-17-producing T helper cells (Th17) and a reduced Treg number are linked to an increased risk of severe PAH. However, recent studies have revealed a complex role of IL-17 in PAH. Higher circulating levels of IL-17A are associated with improved survival in idiopathic patients [11], suggesting a potential protective role in certain patient subgroups. This counterintuitive finding indicates that IL-17 may have context-dependent effects in PAH.

## Microbiome, Immune Response, and PAH

Recent studies have begun to highlight the potential role of the microbiome, encompassing the collective genetic material of bacteria, viruses, and other microorganisms, in modulating immune response in PAH. Understanding how microbiome alterations can contribute to PAH pathogenesis could open new avenues for therapeutic intervention and improve patient outcomes.

Th17 cells play a central role in maintaining epithelial integrity, particularly in mucosal barriers like the gut. Their development and function are closely influenced by microbial signals from the gut microbiota [12]. A diminished Th17–microbiome interaction can disrupt gut epithelial integrity, potentially leading to metabolic disorders, a common comorbidity of PAH (Figure) [13]. The gut epithelium serves as a critical barrier and interface between the host immune system and the vast microbial population residing within the gastrointestinal tract. Integrity of this barrier is essential for preventing the translocation of microbial products into systemic circulation. Recent studies suggest that patients with PAH may exhibit increased intestinal permeability, commonly referred to as “leaky gut” [14]. This increased permeability allows endotoxins such as lipopolysaccharide (LPS) from gram-negative bacteria to enter the bloodstream, potentially triggering systemic inflammation that contributes to the pathogenesis of PAH [15]. Indeed, elevated levels of circulating LPS from the gut microbiome have been observed in PAH patients [16]. Additionally, preliminary data (unpublished data) from our group suggest a decrease in monocyte expression of CD14 in PAH individuals, which may reflect an impaired immune response to endotoxins. The chronic low-grade inflammation resulting from this endotoxemia could exacerbate pulmonary vascular remodeling and hypertension. Furthermore, the pro-inflammatory activity of Th17 cells is heavily influenced by the metabolic environment, including pathways like glycolysis and fatty acid oxidation [17]. These observations collectively point to the possibility that PAH may involve a disrupted microbiome–Th17 interaction. This disruption could be connected to metabolic dysregulation, which reprograms Th17 cells to become more pro-inflammatory and pathogenic.

**Figure.**
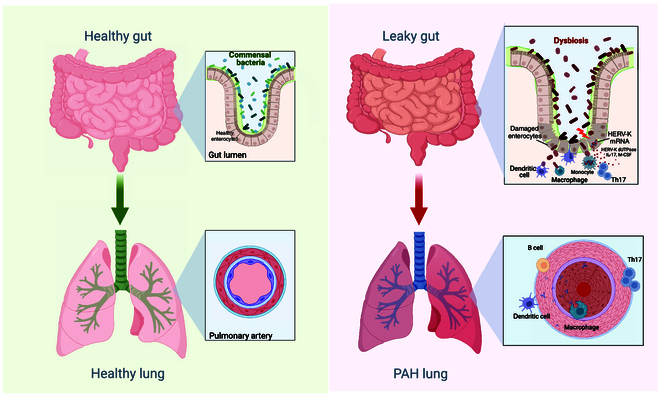
This figure illustrates the hypothesized role of the microbiome in the pathogenesis of pulmonary arterial hypertension (PAH) by comparing a healthy individual (left panel) with a PAH patient (right panel). Left panel (healthy individual): In a healthy state, the gut microbiota is balanced, and the intestinal barrier integrity is maintained. This intact barrier prevents the translocation of microbial components into the systemic circulation. Regulatory immune responses are promoted, and interleukin-17-producing T helper cells (Th17) cells contribute to mucosal immunity without causing excessive inflammation. Right panel (PAH patient with dysbiosis): In PAH, dysbiosis (an unhealthy alteration of the gut microbiome) leads to increased intestinal permeability, known as “leaky gut”. This allows pro-inflammatory microbial components, such as lipopolysaccharide (LPS), to enter the bloodstream. The presence of these microbial products triggers the recruitment of dendritic cells and monocytes. The recruited antigen-presenting cells process and present microbial antigens, leading to the activation of Th17 cells toward a pro-inflammatory phenotype. This shift enhances the production of pro-inflammatory cytokines, contributing to systemic inflammation. The inflammatory environment and immune activation may initiate the transcription of human endogenous retroviruses (HERVs), particularly HERV-K. The expression of HERV-K further stimulates interferon responses and perpetuates inflammation. The systemic inflammation and activated immune cells can migrate to the pulmonary circulation. In the lungs, these factors contribute to vascular remodeling, endothelial dysfunction, and sustained inflammation, which are characteristics of PAH pathology. mRNA, messenger RNA; IL-17, interleukin-17; M-CSF, macrophage colony-stimulating factor.

Dysbiosis of the gut microbiota, an imbalance in microbial composition, has also been implicated in PAH patients [18]. Alterations in the gut microbiome may lead to the production of detrimental metabolites that affect vascular function and immune responses [19]. In light of these findings, fecal microbiota transplantation is being explored in clinical trials as a potential therapeutic strategy for PAH [20], aiming to restore a healthy microbial balance and improve clinical outcomes.

Adding another layer of complexity, an altered human virome is associated with PAH patients. Activation of human endogenous retrovirus K (HERV-K) stimulates an interferon-related antiviral response [21], particularly involving myeloid cells (neutrophils and monocytes), thereby contributing to endothelial–mesenchymal transition [22] and systemic inflammation [23]. Although the precise mechanisms remain unclear, recent research demonstrated that the host’s endogenous retroviruses can be influenced by the microbiota, leading to immune dysregulation that affects tissue homeostasis and vascular inflammation [24]. These prior discoveries collectively suggest a multikingdom dialog between the microbiome and endogenous retroviruses, potentially relevant in PAH pathogenesis. Disruptions in these interactions could contribute to the immune dysregulation and inflammation observed in PAH.

## Future Direction

The emerging evidence, linking the microbiome to PAH pathogenesis, highlights a compelling need for further research to validate these findings. Large-scale, diverse longitudinal cohort studies are essential to determine the prevalence of microbiome-related phenotypes within the PAH patient population. Such studies would help identify specific subtypes of PAH that might benefit from microbiome-targeted interventions such as probiotics, prebiotics, or fecal microbiota transplantation. However, a fundamental challenge lies in accurately identifying these subgroups due to the heterogeneity of the disease.

Understanding the antigenic drivers of the immune response in PAH is another critical area for investigation. It remains unclear whether inflammation is primarily triggered by endogenous retroviruses like HERV-K, bacterial components, or other antigens. Advanced immunological techniques, such as T-cell receptor and B-cell receptor sequencing, could elucidate the specific targets of the adaptive immune system in PAH patients. This approach may reveal whether T and B cells are reacting predominantly to bacterial antigens rather than viral signals, providing valuable insights into the underlying mechanisms of the disease.

Additionally, deciphering the signals that initiate HERV-K transcription in PAH is crucial. Investigating whether microbial factors or other environmental stimuli activate endogenous retroviruses could enhance our understanding of their role in disease pathogenesis. Developing improved cellular and animal models that accurately reflect the complex interactions between the microbiome, immune system, and pulmonary vasculature will be instrumental in this endeavor.

In conclusion, the complex interplay between microbiome, immune dysregulation, and PAH underscores the importance of a multidisciplinary approach to understanding disease mechanisms. As research continues to unravel new disease mechanisms, leveraging the microbiomes’ potential to modulate immune responses may become an innovative strategy to combat PAH and improve patient outcomes.
